# Advances and prospects in deuterium metabolic imaging (DMI): a systematic review of *in vivo* studies

**DOI:** 10.1186/s41747-024-00464-y

**Published:** 2024-06-03

**Authors:** Feng Pan, Xinjie Liu, Jiayu Wan, Yusheng Guo, Peng Sun, Xiaoxiao Zhang, Jiazheng Wang, Qingjia Bao, Lian Yang

**Affiliations:** 1 Department of Radiology, Union Hospital, Tongji Medical College, Huazhong University of Science and Technology, Wuhan, 430022 China; 2 State Key Laboratory of Magnetic Resonance and Atomic and Molecular Physics, Innovation Academy for Precision Measurement Science and Technology, Chinese Academy of Sciences, Wuhan, 430071 China; 3 MSC Clinical & Technical Solutions, Philips Healthcare, Beijing, 100600 China

**Keywords:** Deuterium, Fatty acids, Glycolysis, Magnetic resonance imaging, Magnetic resonance spectroscopy

## Abstract

**Background:**

Deuterium metabolic imaging (DMI) has emerged as a promising non-invasive technique for studying metabolism *in vivo*. This review aims to summarize the current developments and discuss the futures in DMI technique *in vivo*.

**Methods:**

A systematic literature review was conducted based on the PRISMA 2020 statement by two authors. Specific technical details and potential applications of DMI *in vivo* were summarized, including strategies of deuterated metabolites detection, deuterium-labeled tracers and corresponding metabolic pathways *in vivo*, potential clinical applications, routes of tracer administration, quantitative evaluations of metabolisms, and spatial resolution.

**Results:**

Of the 2,248 articles initially retrieved, 34 were finally included, highlighting 2 strategies for detecting deuterated metabolites: direct and indirect DMI. Various deuterated tracers (*e.g.*, [6,6′-^2^H2]glucose, [2,2,2′-^2^H3]acetate) were utilized in DMI to detect and quantify different metabolic pathways such as glycolysis, tricarboxylic acid cycle, and fatty acid oxidation. The quantifications (*e.g.*, lactate level, lactate/glutamine and glutamate ratio) hold promise for diagnosing malignancies and assessing early anti-tumor treatment responses. Tracers can be administered orally, intravenously, or intraperitoneally, either through bolus administration or continuous infusion. For metabolic quantification, both serial time point methods (including kinetic analysis and calculation of area under the curves) and single time point quantifications are viable. However, insufficient spatial resolution remains a major challenge in DMI (*e.g.*, 3.3-mL spatial resolution with 10-min acquisition at 3 T).

**Conclusions:**

Enhancing spatial resolution can facilitate the clinical translation of DMI. Furthermore, optimizing tracer synthesis, administration protocols, and quantification methodologies will further enhance their clinical applicability.

**Relevance statement:**

Deuterium metabolic imaging, a promising non-invasive technique, is systematically discussed in this review for its current progression, limitations, and future directions in studying *in vivo* energetic metabolism, displaying a relevant clinical potential.

**Key points:**

• Deuterium metabolic imaging (DMI) shows promise for studying *in vivo* energetic metabolism.

• This review explores DMI’s current state, limits, and future research directions comprehensively.

• The clinical translation of DMI is mainly impeded by limitations in spatial resolution.

**Graphical Abstract:**

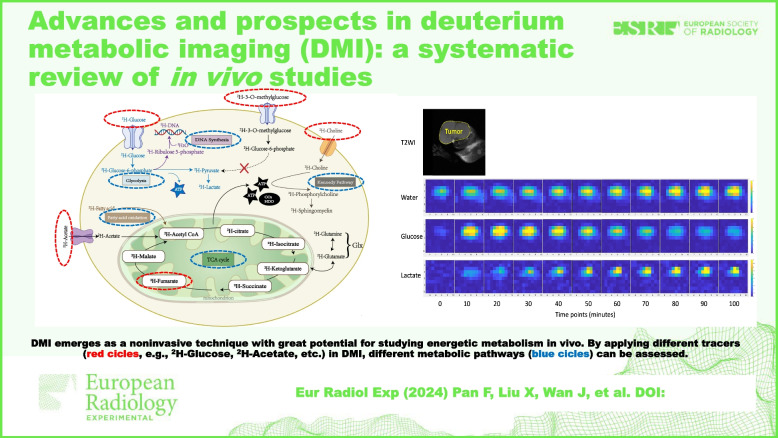

**Supplementary Information:**

The online version contains supplementary material available at 10.1186/s41747-024-00464-y.

## Background

Metabolic imaging is a class of non-invasive techniques that enable *in vivo* visualization and quantification of metabolic processes such as glucose uptake, glycolysis, and tricarboxylic acid (TCA) cycle [1, 12, 14, 15, 23, 25, 54, 59, 68, 75]. It can detect malignant tumors with high sensitivity and specificity by imaging their increased metabolic rates for glucose, amino acids, or lipids; additionally, it offers valuable insights into risk stratification and treatment response evaluation of malignant tumors [1, 12, 14, 15, 23, 25, 54, 59, 68, 75]. So far, many metabolic imaging techniques have emerged, including fluorine-18-fluorodeoxyglucose positron emission tomography, ^1^H-magnetic resonance spectroscopic imaging (^1^H-MRSI), chemical shift saturation transfer, hyperpolarized-^13^C-magnetic resonance imaging (hyperpolarized-^13^C-MRI), and magnetic resonance (MR)-based deuterium metabolic imaging (DMI) [9, 14, 16, 25, 30, 36, 54, 58, 75].

Fluorine-18-fluorodeoxyglucose positron emission tomography is widely used in oncology for tumor staging, prognostic prediction, and treatment response evaluation [1, 5, 9, 33, 78]. However, it is limited by radiation exposure (about 25 mSv for each examination) and incapability in monitoring downstream metabolism except for glucose intake [6, 33]. On the other hand, MRI and MRS, such as ^1^H-MRS/MRSI and chemical shift saturation transfer, can monitor metabolism *in vivo* without exposing patients to ionizing radiation [30, 36, 54]. However, these techniques can only be applied to observe the steady-state metabolism with complicated peak overlaps between different molecules (*e.g.*, water, lipids) [54, 70]. To address these issues, a hyperpolarized MRI technique has been developed in the past decade [14, 23, 25, 59, 68, 75]. By utilizing the dynamic nuclear polarization technique, the MR signal of certain nuclei, such as ^13^C, can be enhanced for more than 10,000 folds [26, 51]. Hyperpolarized MRI enables the visualization of metabolic pathways in real-time following the administration of exogenous hyperpolarized-^13^C-labeled tracers (*e.g.*, hyperpolarized-^13^C-pyruvate, hyperpolarized-^13^C-fumarate) [23, 26, 59, 75]. However, the clinical translation of hyperpolarized MRI is slow due to the short-term enhanced signal (1–2 min) and the high cost of the equipment.

DMI is a relatively new MR-based imaging technique that utilizes exogenous non-radioactive and biocompatible deuterium-labeled metabolic tracers such as [6,6′-^2^H_2_]glucose and [^2^H_3_]acetate to visualize different metabolic pathways *in vivo* [15, 16, 58]. Compared to the aforementioned metabolic imaging techniques, DMI offers several distinct advantages including non-ionizing radiation, stable isotope labeling, biochemical safety, and relatively simple techniques [15, 16, 44, 58]. Compared with hyperpolarized-^13^C-MRI, DMI can be applied to observe a longer-term metabolic process (more than hours) [16]. Although deuterium has a lower gyromagnetic ratio than the ^1^H proton (6.536 MHz/T *versus* 42.577 MHz/T), it has a rapid longitudinal relaxation that enhances its detection [16, 81]. Unlike ^1^H-MRSI, DMI does not require additional water suppression due to the low abundance of natural deuterated water (0.0115%, 10.12 mM), leading to a low specific absorption rate of radiofrequency power [15, 16, 44]. Moreover, because of the reduced coupling effects of deuterium, the spectrum in DMI is much simpler to interpret than that of ^1^H that has complex peak-splitting patterns [15, 16]. The combination of the low natural abundance of deuterium and the selective deuterium-labeling of metabolites by the tracer contribute to the further simplification of the spectrum in DMI. So far, many outstanding reviews or comments have been published on the applications of DMI [12, 16, 53, 58, 67, 76, 79, 82]. To avoid repetition, this systematic review aims to summarize the current development of the DMI technique *in vivo*, outline present limitations, and discuss the potential research and development directions for the future.

## Methods

### Strategy of literature review

A systematic literature review was performed to summarize the deuterium metabolic MRI or spectroscopy *in vivo* based on the PRISMA 2020 statement [55]. The PubMed database was used for the MeSH term search for previously published studies on deuterium metabolic MR imaging or spectroscopy from 1 January 2003 to 13 May 2023 (encompass earlier works that laid the foundation for subsequent advancements). The search strategy is described in Additional file 1: Table S1. A reference check was also performed. The inclusion criteria include the following: (1) original articles published with specific technique descriptions of MR imaging or spectroscopy involving deuterium detection to explore the metabolic processes *in vivo* and (2) studies were performed in living humans or animals, excluding studies performed on cell lines or isolated samples from humans or animals. Studies were selected for inclusion by two authors, who have 15 years of experience in diagnostic radiology and 15 years of experience in MR experiments. Decisions were made by consensus among all authors.

### Data extraction and summarization

After conducting the literature review, a comprehensive extraction of specific technical details (including study subjects, examined body parts, target diseases/specific physiological conditions, deuterated tracers, administration dose/route, and MR equipment/scanning parameters) was performed from the relevant articles. Additionally, potential applications, such as examination purposes and metabolic biomarkers, were collected. Two authors (FP and PS) performed the data extraction from all included studies. Subsequently, several key topics were summarized, including strategies of deuterated metabolites detection, deuterium-labeled tracers and corresponding metabolic pathways *in vivo*, potential clinical applications, routes of tracer administration, quantitative evaluations of metabolisms, and spatial resolution.

## Results

**Fig. 1:**
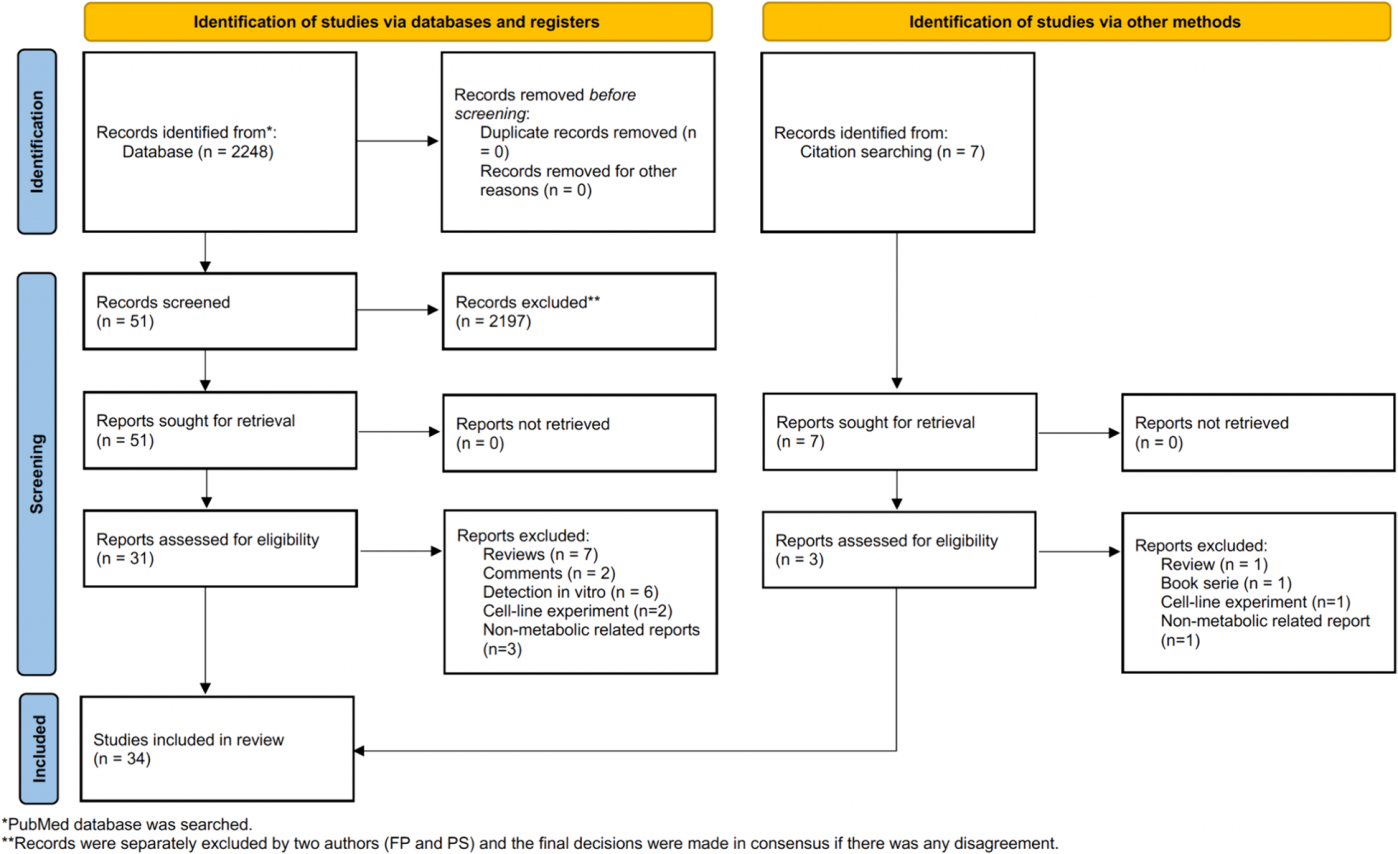
PRISMA 2020 flow diagram of the retrieved database

The initial literature search resulted in a total of 2,248 articles. Following the screening process, 2,217 articles were excluded based on the inclusion criteria. Additionally, 3 articles were identified through citation searching. Finally, 34 published articles were involved (Table 1). The details of the database retrieval are shown in Fig. 1.

**Table 1:** The 34 included articles

First author [reference number]	Year	Deuterated tracers	Target living subjects in the study	Examined body parts by DMI	Target diseases or specific physiological conditions	MR equipment
Buxbaum [8]	2017	^2^H_2_O	Mice after hematopoietic stem cell transplantation	Liver	Chronic graft-versus-host disease	9.4 T^a^
Lu [44]	2017	[6,6′-^2^H2]glucose	Healthy rats	Brain	Deep anesthesia; morphine administration	16.4 T^a^
De Feyter [15]	2018	[6,6′-^2^H2]glucose; [2,2,2′-^2^H3]acetate	Human patients; tumor-bearing rats	Brain; liver	Glioblastoma; gliosarcoma	11.7 T (for rats)^a^; 4 T (for humans)^a^
de Graaf [18]	2020	[6,6′-^2^H2]glucose	Healthy human volunteers	Brain	–	4 T^a^; 7 T^a^
Kreis [37]	2020	[6,6′-^2^H2]glucose	Tumor-bearing rats	Subcutaneous tissue	Lymphoma	9.4 T^a^
Rich [60]	2020	[6,6′-^2^H2]glucose; [2,2,2′-^2^H3]acetate	Tumor-bearing rats	Brain	Glioblastoma	9.4 T^b^
Riis-Vestergaard [61]	2020	[6,6′-^2^H2]glucose	Healthy rats	Interscapular brown adipose tissue depot	Cold acclimation	9.4 T^a^
De Feyter [17]	2021	[6,6′-^2^H2]glucose	Healthy rats	Liver	–	11.7 T^a^
Hartmann [27]	2021	Deuterated 3-O-methylglucose	Tumor-bearing rats	Hind leg	Breast cancer	7 T^a^
Hesse [28]	2021	[2,3-^2^H2]fumarate	Tumor-bearing mice	Subcutaneous tissue of flank	Lymphoma; breast cancer; colorectal cancer	7 T^1^
Li Y [41]	2021	[6,6′-^2^H2]glucose	Tumor-bearing rats	Brain	Gliosarcoma	16.4 T^a^
Mahar [46]	2021	[^2^H7]glucose	Healthy rats	Brain	–	11.1 T^a^
Markovic [47]	2021	[6,6′-^2^H2]glucose	Tumor-bearing mice	Pancreas	Pancreatic ductal adenocarcinoma	15.2 T^a^
Markovic [48]	2021	[6,6′-^2^H2]glucose	Pregnant mice	Fetus and placenta	Preeclampsia	15.2 T^a^
Peters [56]	2021	[6,6′-^2^H2]glucose	Tumor-bearing mice	Pancreas	Pancreatic ductal adenocarcinoma	15.2 T^a^
Ruhm [62]	2021	[6,6′-^2^H2]glucose	Healthy human volunteers	Brain	–	9.4 T^1^
Veltien [72]	2021	[^2^H9]choline; [^2^H9]choline + [6,6′-^2^H2]glucose	Tumor-bearing mice	Subcutaneous tissue of flank	Renal carcinoma	11.7 T^a^
von Morze [73]	2021	[6,6′-^2^H2]glucose	Healthy rats	Brain	–	4.7 T^a^
Wang [74]	2021	[6,6′-^2^H2]glucose; [2,2,2′-^2^H3]acetate	Healthy rats	Heart	–	16.4 T^a^
Batsios [2]	2022	[U-^2^H]pyruvate	Tumor-bearing mice	Brain; subcutaneous tissue	Glioblastoma; oligodendroglioma; hepatocellular carcinoma	14.1 T^a^; 3 T^a^
Cember [10]	2022	[6,6′-^2^H2]glucose	Healthy human volunteers	Brain	–	7 T^b^
Ge [24]	2022	[6,6′-^2^H2]glucose	Tumor-bearing mice	Brain	Glioblastoma	11.74 T^a^
Hesse [29]	2022	[2,3-^2^H2]fumarate	Tumor-bearing mice	Subcutaneous tissue of flank	Lymphoma	7 T^a^
Kaggie [32]	2022	[6,6′-^2^H2]glucose	Healthy human volunteers	Brain	–	3 T^a^
Liu [43]	2022	[6,6′-^2^H2]glucose	Healthy human volunteers	Brain	–	4 T^a^
Meerwaldt [50]	2022	[6,6′-^2^H2]glucose	Mice with ischemic stroke	Brain	Stroke	9.4 T^a^
Niess [52]	2022	[6,6′-^2^H2]glucose	Healthy human volunteers	Brain	–	3 T^b^
Serés Roig [65]	2022	[6,6′-^2^H2]glucose	Healthy human volunteers	Brain	–	7 T^a^
Simões [66]	2022	[6,6′-^2^H2]glucose	Tumor-bearing mice	Brain	Glioblastoma	9.4 T^a^
Taglang [69]	2022	[6,6′-^2^H2]glucose	Tumor-bearing mice	Brain	Astrocytoma	14.1 T^a^
Zhang [81]	2022	[6,6′-^2^H2]glucose	Tumor-bearing mice	Subcutaneous tissue of scapula	Melanoma	9.4 T^a^
Zou [83]	2022	[6,6′-^2^H2]glucose; [2,3,4,6,6′-^2^H5]glucose	Tumor-bearing rats	Brain	Glioma	9.4 T^a^
Bednarik [3]	2023	[6,6′-^2^H2]glucose	Healthy human volunteers	Brain	–	7 T^b^
Ip [31]	2023	[^2^H9]choline	Tumor-bearing rats	Brain	Glioblastoma	11.74 T^a^

*DMI* Deuterium metabolic imaging, *MR* Magnetic resonance

^a^Direct DMI was performed in the study

^b^Indirect DMI was performed in the study

### Strategies of deuterated metabolites detection: direct and indirect DMI

**Fig. 2:**
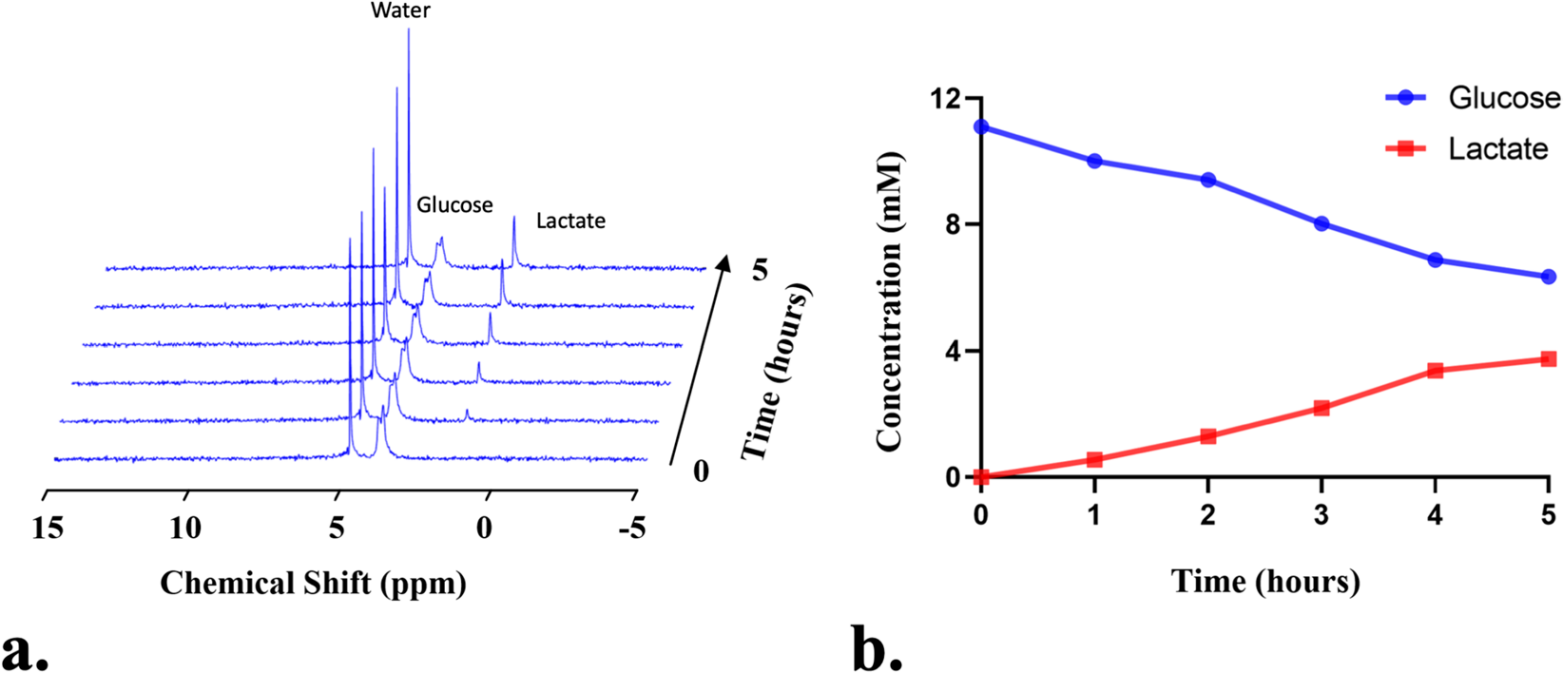
A representative ^2^H-MRS obtained from the liquid cultured medium for a tumor cell line (MC38 murine colon adenocarcinoma) after being exposed to [6,6′-^2^H2]glucose at different time points (**a**). Over time, the ^2^H-labeled glucose level gradually decreased but the ^2^H-labeled lactate gradually increased indicating a typical Warburg effect of the malignant cell line (**b**). Figures were provided by author QB. All experiment was performed in Bruker 500-MHz MR scanner

**Fig. 3:**
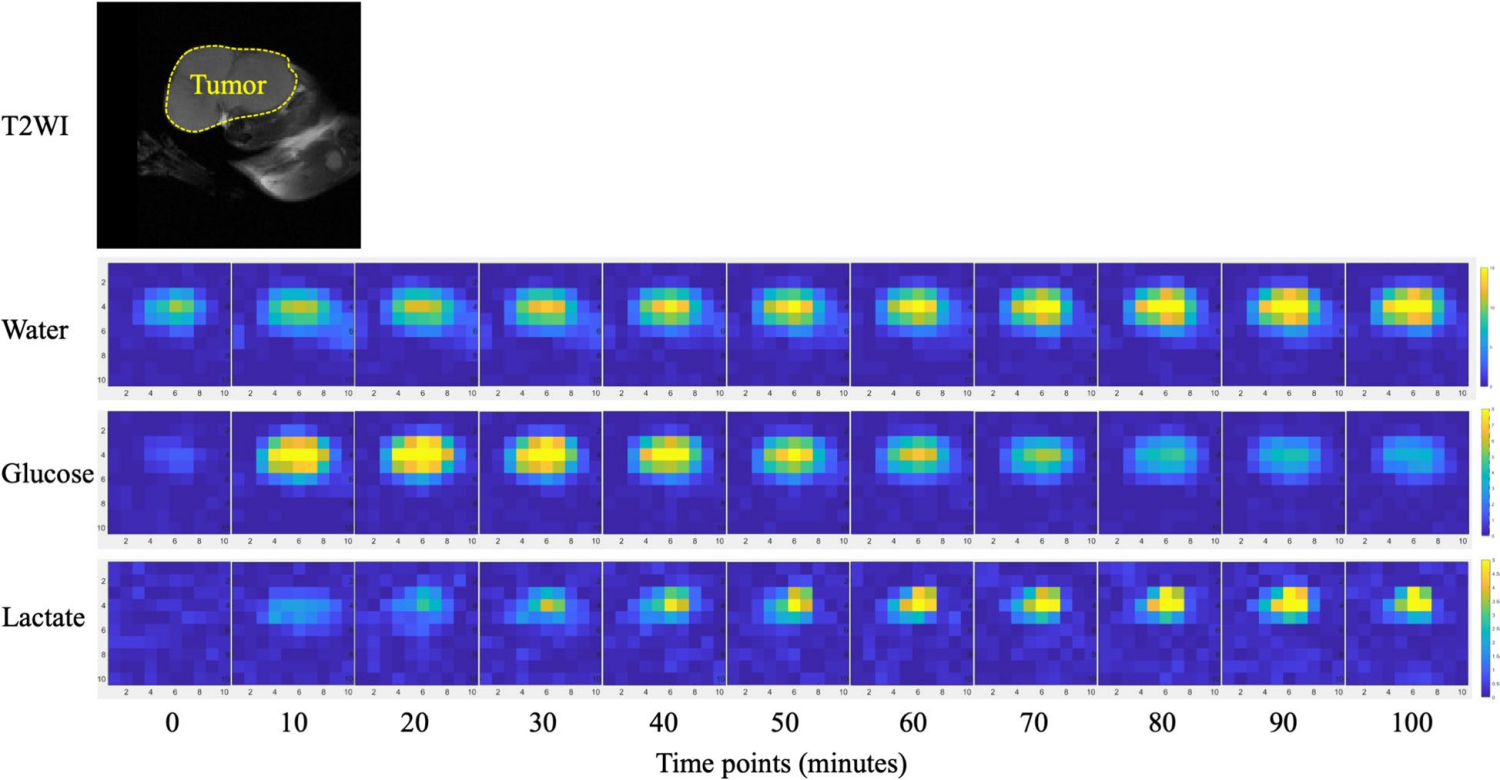
A representative deuterium metabolic imaging visualizing the Warburg effect in an MC38 tumor inoculated C57BL/6 male mouse after a bolus intravenous injection of [6-6′-^2^H2]glucose (1.0 g/kg mouse weight). Over time, the ^2^H-labeled glucose level gradually decreased, but the ^2^H-labeled lactate gradually increased in the tumor region, indicating a typical Warburg effect of the malignancy. Figures were provided by author QB. All experiment was performed in Bruker 9.4-T MR scanner

**Fig. 4:**
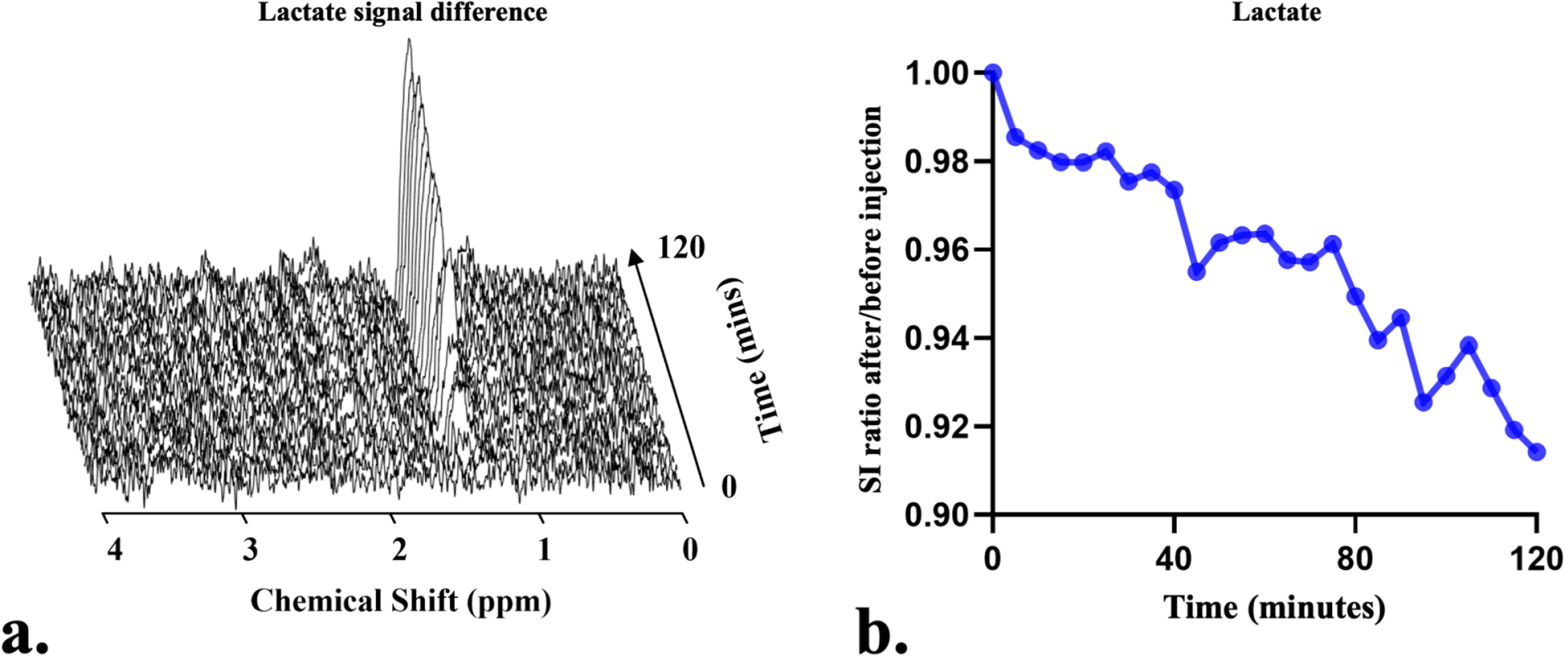
An exemplary ^1^H-MR difference spectrum from a voxel (3 mm × 3 mm × 3 mm) of MC38 tumor in the C57BL/6 male mouse thigh with an illustration of the dynamic change of lactate signals after an intravenous infusion of [6,6′-^2^H2]glucose (1.0 g/kg mouse weight) (**a**). Over time, the signal difference of lactate gradually increased with a gradual decrease of the lactate signal intensity (SI) ratio after/before tracer injection (**b**). Figures were provided by author QB. All experiment was performed in 9.4-T MR scanner

From the included articles (Table 1), detecting deuterium-labeled metabolites by DMI typically involves 2 strategies: direct (30 studies, including 25 animal studies and 6 human studies, with 1 study conducted in both animals and humans) and indirect (4 studies, including 3 human studies and 1 animal study) DMI. In the former strategy, ^2^H-MRS/MRSI sequences were applied to selectively excite the deuterium nuclei and specifically capture the deuterium-labeled metabolite signals over time (Figs. 2 and 3) [15, 62, 65, 73, 81]. In the latter strategy, the deuterium-labeled metabolites were not excited but detected indirectly through the corresponding ^1^H-MR signal decrease, due to the chemical exchange between hydrogen and deuterium atoms following the administration of deuterium-labeled tracers (Fig. 4) [3, 10, 52, 60].

### Deuterium-labeled tracers and corresponding metabolic pathways *in vivo*

**Fig. 5:**
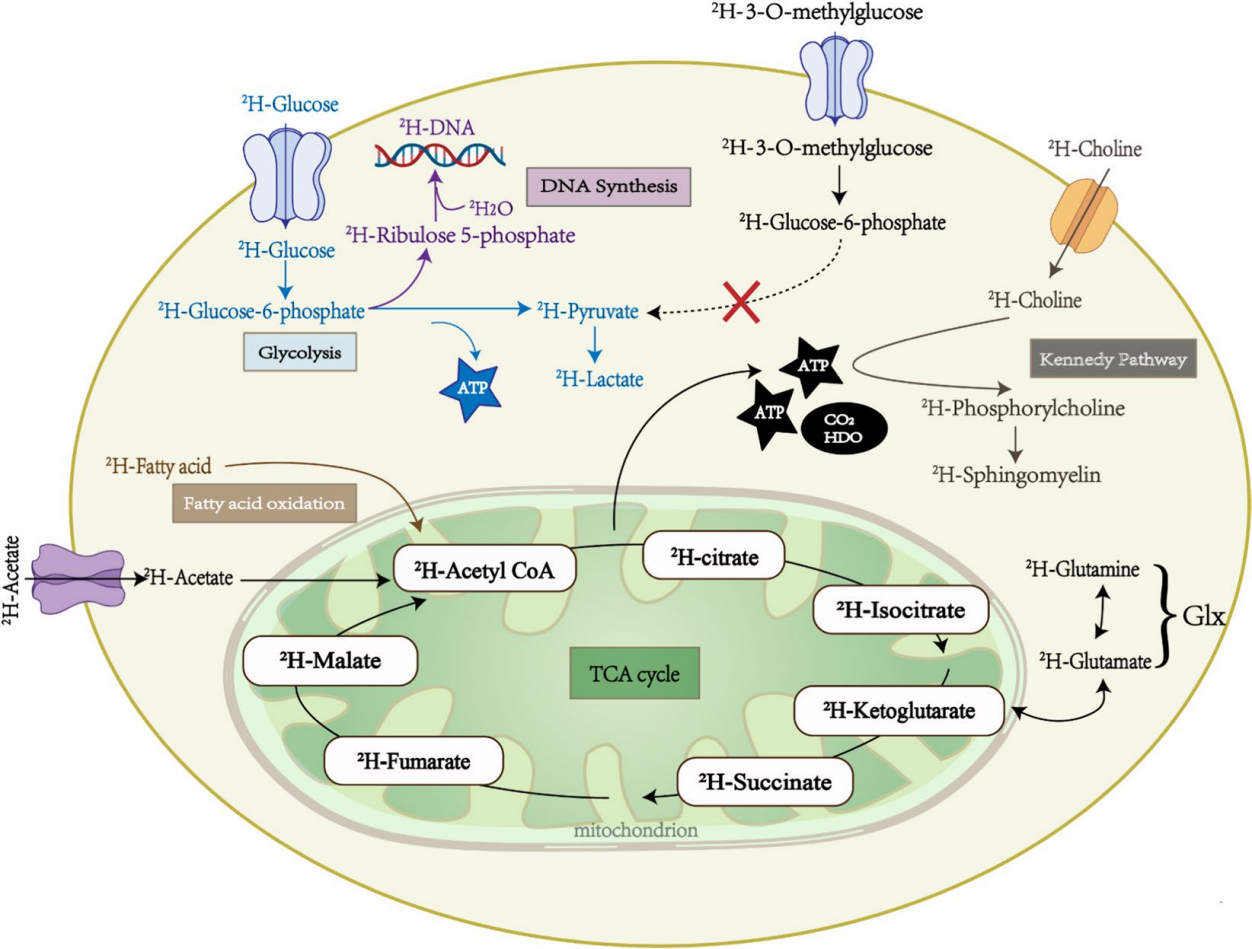
An overview of cellular metabolic key pathways detected by deuterium metabolic imaging: glycolysis, TCA cycle, fatty acid oxidation, deoxyribonucleic acid synthesis, and Kennedy pathway. *ATP* Adenosine triphosphate, *CO*_*2*_ Carbon dioxide, *GLUT* Glucose transporter, *Glx* Glutamine and glutamate, *HDO* Semiheavy water, *TCA* Tricarboxylic acid

Deuterated tracers used in DMI are always the metabolites that are part of the cellular regular metabolism pathways [34]. They contain deuterium atoms and can be metabolized like their non-labeled counterparts. When administered, these tracers can be incorporated into the corresponding metabolic pathways (*e.g.*, glycolysis, TCA cycle, fatty acid oxidation) and metabolized into downstream deuterated compounds which can be detected and quantified by MRS/MRSI (Fig. 5) [34]. Although a high-dose deuterium intake is harmful, the tracer doses widely employed in DMI, especially within the range of 0.6–0.8 g/kg [6,6′-^2^H2]glucose, have been extensively demonstrated to be non-toxic *in vivo* through previous studies [3, 10, 15, 18, 32, 43, 52, 62, 65].

Most tracers were produced from the deuteration of protonated metabolites that are associated with energy metabolism or cellular proliferation, including glucose, pyruvate, acetate, fumarate, and choline [2, 3, 10, 15, 17, 18, 24, 28, 29, 31, 32, 37, 41, 43, 44, 46–48, 50, 52, 56, 60–62, 65, 66, 69, 72–74, 81, 83]. For instance, [6,6′-^2^H2]glucose, as a mostly used metabolic tracer, can be used to detect downstream deuterated products, including glutamate, glutamine, lactate, and semiheavy water (Fig. 5) [2, 3, 10, 15, 17, 18, 24, 28, 29, 31, 32, 37, 41, 43, 44, 46–48, 50, 52, 56, 60–62, 65, 66, 69, 72–74, 81, 83]. Among these downstream metabolites, glutamate and glutamine (Glx) can be regarded as products from TCA cycle and lactate from glycolysis [2, 3, 10, 15, 17, 18, 24, 28, 29, 31, 32, 37, 41, 43, 44, 46–48, 50, 52, 56, 60–62, 65, 66, 69, 72–74, 81, 83]. By measuring the incorporation of deuterium into these products, the rates of glycolysis and TCA cycle can be estimated [2, 3, 10, 15, 17, 18, 24, 28, 29, 31, 32, 37, 41, 43, 44, 46–48, 50, 52, 56, 60–62, 65, 66, 69, 72–74, 81, 83]. However, the low deuterium enrichment of [6,6′-^2^H2]glucose restricts its signal intensity [15]. Therefore, scientists are interested in developing novel glucose tracers with more deuterium atoms within the molecules, such as [2,3,4,6,6′-^2^H5]-d-glucose and [^2^H7]glucose [15, 45, 46, 83]. These tracers may have some advantages over [6,6′-^2^H2]glucose, such as higher signal intensities and enhanced tolerability to label loss [15, 45, 46, 83].

As examples for other tracers, fatty acid oxidation involves the breakdown of fatty acids to generate acetyl-coenzyme A, which can enter the TCA cycle for further metabolism; so, by administering a deuterium-labeled fatty acid [2,2,2′-^2^H3]acetate, the fatty acid oxidation can be traced by the products of Glx (Fig. 5) [15, 60, 74]. Similarly, [2,3-^2^H2]fumarate, a precursor of malate, and [U-^2^H]pyruvate, a precursor of lactate, can be specifically used to measure the rates of TCA cycle and glycolysis, respectively (Fig. 5) [2, 28, 29].

In addition, some essential materials for cell proliferation can also be deuterated as tracers in DMI, such as [^2^H9]choline and heavy water (Fig. 5) [8, 31, 72]. Choline is a precursor for phosphatidylcholine, a major component of cell membranes produced in the Kennedy pathway. By using [^2^H9]choline as a tracer, DMI can image and quantify the total pool of choline *in vivo*, which can reflect the cellular proliferation activity [31, 72]. Exogenous heavy water administrated into the body water pool can also be used as a tracer, which can be incorporated into various macromolecules such as lipids, proteins, and deoxyribonucleic acid [7, 8, 20, 42]. However, most synthesized molecules such as lipids and proteins will gradually lose deuterium labels over time owing to the energy metabolism except de novo deoxyribonucleic acid; thus, measuring long-term deuterium enrichments can help to quantify the de novo deoxyribonucleic acid which can serve as a proxy indicator for cell proliferation [7, 8, 20].

Moreover, another tracer type is the non-metabolizable analog of metabolic precursors. For example, deuterated 3-O-methylglucose is a glucose analog that is taken up by cells but not metabolized further, thus specifically reflecting the glucose uptake [27].

### Potential clinical applications

As a novel and still evolving technique, most *in vivo* DMI studies have been performed in animal brains and gliomas because of relatively static organs, and the results from these studies have shown great potential for a range of clinical applications (Table 2) [2, 15, 27, 31, 37, 41, 47, 56, 60, 66, 69, 72, 81, 83]. The most common application of DMI is to diagnose malignant tumors by detecting abnormal metabolic changes in the material requirement, glycolysis, and TCA cycle [2, 15, 27, 37, 41, 47, 56, 60, 66, 69, 72, 81, 83]. Because most malignancies have increased demands for proliferation, an elevated consumption or uptake of different materials (including glucose and choline) can be detected by DMI following administrating tracers [71]. Moreover, compared to normal tissue, malignant tumors present a so-called Warburg effect, presenting significantly increased glycolysis (elevated lactate) and reduced TCA cycle activity (decreased Glx) [35, 71]. This metabolism alteration can be specifically detected after applying various deuterated tracers (including [6,6′-^2^H2]glucose, [U-^2^H]pyruvate, and [2,3-^2^H2]fumarate) in DMI, which demonstrates higher lactate levels, lower Glx synthesis/flux rates, higher lactate/Glx ratios, or lower fumarate/malate conversion rate [2, 15, 41, 60, 66, 69, 83]. Specifically, the biomarker “lactate/Glx ratio,” as a rational indicator of the Warburg effect, is considered sensitive to detect solid malignancies located in organs with high-glucose intakes and consumptions, such as the brains and kidneys [11, 15, 37, 41, 57, 83]. In these organs, where positron emission tomography is limited due to the possible similarity in glucose uptake between tumors and background tissues, the lactate/Glx ratio serves as a valuable alternative for assessing metabolic abnormalities associated with malignancies.

**Table 2:** Potential applications of DMI *in vivo*

Potential applications	Deuterated tracers	Biomarkers	Explanations
Oncological diagnosis			
Breast cancer [27]	Deuterated 3-O-methylglucose [27]	Deuterated 3-O-methylglucose uptake	Tumor tissue has a higher uptake of glucose, as well as glucose analog such as deuterated 3-O-methylglucose.
Gliomas (astrocytoma, oligodendroglioma, and glioblastoma) [2, 15, 31, 41, 60, 66, 69, 83]	[6,6′-^2^H2]glucose and [2,3,4,6,6′-^2^H5]-D-glucose [15, 41, 66, 69, 83]	Maximum glucose consumption rate, lactate and Glx synthesis levels/flux rates, and lactate/Glx ratio	Due to the high proliferation activity, malignant tumor has a higher energy requirement of glucose leading to a significantly increased glucose consumption. Besides, because of the Warburg effect which is a typical feature of malignant tumor, glycolysis predominates the energy supply for the tumor cells; as a result, more lactate is produced and the TCA cycle is inhibited, as well as the Glx production. So, a typical malignant tumor presents an elevated lactate level, a decreased Glx level, and an increased lactate/Glx ratio.
	[U-^2^H]-pyruvate [2]	Lactate synthesis level	Pyruvate can be converted into lactate or enter the TCA cycle. But in malignancies, because of the Warburg effect, the conversion of pyruvate to lactate, or the so-called glycolysis process, is predominant. Thus, the malignant tumor presents a significantly higher lactate level.
	[2,2,2′-^2^H3]acetate [15, 60]	Glx synthesis level	Acetate can be considered a surrogate for fatty acid oxidation. After being converted to acetyl-coenzyme A, acetate can join the TCA cycle to produce energy. In the TCA cycle, acetyl-coenzyme A occurs fast exchange with another molecule called alpha-ketoglutarate, which can then become Glx that can be detected by MRI. So, the Glx level can reflect the cellular TCA cycle activity, which is significantly suppressed in malignancies.
	[^2^H9]choline [31]	Total choline uptake	Choline is a component of phospholipids, which are essential for the formation and maintenance of cell membranes. Tumor cells have increased choline uptake and metabolism due to their high demand for membrane synthesis and growth. Thus, the malignant tumor presents a higher choline uptake than normal tissue.
Hepatocellular carcinoma [2]	[U-^2^H]-pyruvate [2]	See above	See above
Lymphoma [37]	[6,6′-^2^H2]glucose [37]	See above	See above
Melanoma [2, 81]	[6,6′-^2^H2]glucose [81]	See above	See above
	[U-^2^H]-pyruvate [2]	See above	See above
Neuroblastoma [2]	[U-^2^H]-pyruvate [2]	See above	See above
Pancreatic ductal adenocarcinoma [47, 56]	[6,6′-^2^H2]glucose [47, 56]	See above	See above
Renal carcinoma [72]	[^2^H9]choline [72]	See above	See above
	[6,6′-^2^H2]glucose [72]	See above	See above
Early response to anticancer treatment			
Gliomas after chemotherapy, dichloroacetate, TERT inhibitor, or PARP inhibitor; lymphoma after chemotherapy [2, 15, 37, 69]	[6,6′-^2^H2]glucose [15, 37, 69]	Maximum glucose consumption rate, lactate/water ratio, lactate/glucose ratio, lactate/Glx ratio	After effective treatment, the Warburg effect can be converted resulting in a significant increase in oxidative metabolism when compared to aerobic glycolysis. Thus, a decreased lactate/Glx ratio can reflect an effective response at an early stage.
	[U-^2^H]-pyruvate [2]	Lactate synthesis	After effective treatment, the glycolysis was inhibited leading to a significantly reduced lactate production at an early time point before a change in tumor size.
Breast cancer, colorectal cancer, and lymphoma after chemotherapy [28]	[2,3-^2^H2]fumarate [28]	Fumarate to malate conversion	When the cellular plasma membrane is damaged after effective anti-tumor treatment, fumarate can enter the cell quickly and react with fumarase to produce malate. So, an increased malate/fumarate ratio indicates tumor necrosis after treatment and in principle should be more sensitive for detecting cell death than imaging techniques that rely on a morphological change.
Residual and necrotized tumor evaluation			
Glioblastoma after radiotherapy [24]	[6,6′-^2^H2]glucose [24]	Lactate and Glx synthesis levels and lactate/Glx ratio	Malignant tumor shows a highly significant dominance of aerobic glycolysis (Warburg effect) but radiation necrotized tumor presents a conversion to oxidative respiration (predominant TCA cycle). Thus, recovered lactate and Glx levels in the tumor indicate a necrotized change after radiotherapy.
Diagnosis of other diseases			
Chronic graft-versus-host disease [8]	^2^H_2_O [8]	Deuterium accumulation	Deuterium from ^2^H_2_O can incorporate into cellular deoxyribonucleic acid through the constitutive *de novo* nucleotide synthesis pathway. Following hematopoietic stem cell transplantation, rapidly proliferating T cells (Tem subset) can be found in the chronic graft-versus-host disease condition. This urges the nucleotide synthesis, resulting in an elevated deuterium accumulation *in vivo*.
Preeclampsia [48]	[6,6′-^2^H2]glucose [48]	Lactate synthesis level	Preeclampsia is caused by constriction of the systematic arterial vessels, resulting in hypoxic conditions of fetoplacental units. Afterwards, anaerobic glycolysis is enhanced in the placenta and fetus demonstrating a significantly elevated lactate level.
Rehabilitation evaluation			
Ischemic stroke [50]	[6,6′-^2^H2]glucose [50]	Lactate and Glx synthesis	After ischemic stroke, normal oxidative metabolism is replaced by glycolysis resulting in cerebral lactate accumulation and Glx depletion. Monitoring the changes of lactate and Glx levels in the stroke region can help to evaluate cerebral recovery over time.
Specific physiological conditions			
Brown adipose tissue activity under cold acclimation [61]	[6,6′-^2^H2]glucose [61]	Glucose uptake	Activated brown adipose tissue is essential to combat obesity and metabolic syndrome *in vivo*, because it brings an increased energy expenditure. Thus, activated brown adipose tissue has a significantly higher glucose uptake.
Deep anesthesia and analgesia [44]	[6,6′-^2^H2]glucose [44]	Glucose consumption rate, TCA flux rate	Different physiopathological states (*e.g.*, deep anesthesia and analgesia conditions) can cause altered metabolic activity of the brain including glucose consumption and oxidative metabolism.

*DMI* Deuterium metabolic imaging, *Glx* Glutamine and glutamate, *TCA* Tricarboxylic acid, *tCho* Total choline pool, *TERT* Telomerase reverse transcriptase, *PARP* Poly adenosine diphosphate-ribose polymerase

DMI has also presented great potential in estimating residual tumors and early response after various anticancer treatments (*e.g.*, chemotherapy, targeted therapy, radiotherapy) (Table 2) [2, 15, 24, 28, 37, 69]. From previous studies, DMI presented a higher sensitivity in identifying tumoral necrosis and residue than conventional imaging techniques that mainly rely on morphological changes [2, 15, 24, 28, 37, 69]. A necrotized tumor after treatments first demonstrated a significantly declined Warburg effect, accompanied by a recovered TCA cycle activity and suppressed glycolysis, before the reduction of glucose intake and following morphological necrosis [15, 24, 37, 69]. Thus, DMI can also be applied in the early treatment-response evaluation with a potentially higher sensitivity than conventional methods; for example, previous studies revealed that early tumoral necrosis presented a significantly increased fumarate/malate conversion rate (recovered TCA cycle activity) after administrating [2,3-^2^H2]fumarate in DMI [28, 75].

Moreover, some animal studies found that DMI can also reflect various metabolic abnormalities under other diseases or physiological conditions, such as chronic graft-versus-host disease after hematopoietic stem cell transplantation, preeclampsia, ischemic stroke, cold acclimation, deep anesthesia, and analgesia condition (Table 2) [8, 44, 48, 50, 61]. Although these experiments were performed on animals, they can also be extended to human beings [8, 44, 48, 50, 61].

### Routes of tracer administration: oral, intravenous bolus/infusion, and intraperitoneal

In DMI, deuterated tracers can be administered in several ways (Table 3): oral intake, intravenous (IV) bolus injection, IV infusion, intraperitoneal (IP) bolus injection, IP infusion, applying a bolus variable infusion protocol, and IV bolus injection followed by oral intake.

**Table 3:** Different administration routes of deuterated tracers *in vivo*

Administration routes	Study subjects	Tracers	Tracer dose	Periods (approx.)
Oral intake	Human [3, 10, 15, 18, 32, 43, 52, 62, 65]	[6,6′-^2^H2]glucose	0.60–0.80 g/kg (max, 55–60 g)	NA
	Mice [29]	[2,3-^2^H2]fumarate	2.0 g/kg	NA
IV bolus injection	Mice [47, 48, 56, 66, 69]; rats [37, 41, 44, 46, 61, 73, 83]	[6,6′-^2^H2]glucose; [2,3,4,6,6′-^2^H5]glucose; [^2^H7]glucose	1.0–4.0 g/kg	1–2 min
	Rats [27]	Deuterated 3-O-methylglucose	0.89 g/kg	1 min
	Mice [2]	[U-^2^H]pyruvate	0.45 g/kg	NA
	Mice [72]	[^2^H9]choline	0.05 g/kg	20 s
IV infusion	Mice [24, 48, 50]; rats [15, 17, 74]	[6,6′-^2^H2]glucose	1.5–2.3 g/kg	1–2 h^a^
	Mice [28]	[2,3-^2^H2]fumarate	1.0 g/kg	20 min
	Rats [15, 74]	[2,2,2′-^2^H3]acetate	1.0–2.0 g/kg; 0.5 or 1.0 g/rat	0.3–2 h
IP bolus injection	Mice [81]	[6,6′-^2^H2]glucose	2.0 or 6.0 g/kg	NA
IP infusion	Rats [15, 17]	[6,6′-^2^H2]glucose	1.50–1.95 g/kg	2 h
	Rats [15, 74]	[2,2,2′-^2^H3]acetate	2.0 g/kg	2 h
A bolus variable infusion protocol^b^	Rats [60]	[6,6′-^2^H2]glucose	1.95 g/kg	1 h
	Rats [60]	[2,2,2′-^2^H3]acetate	2.0 g/kg	1 h
	Rats [31]	[^2^H9]choline	0.376 g/kg	1 h
IV bolus injection followed by oral intake^c^	Mice [8]	^2^H_2_O	35 mL/kg, 8% (v/v) [20]	1 week

*NA* Not applicable, *IP* Intraperitoneal, *IV* Intravenous

^a^There is an exception in that one study reported an 8-min IV infusion [74]

^b^Briefly, it is a three-step bolus-continuous infusion protocol, in which animals received an IV bolus injection of the deuterated tracer (about one-sixth of the total dose) over 15 s, followed by a gradual reduction of infusion until a constant infusion rate was reached for the remainder of the experiment [31, 60]

^c^Briefly, an initial IP bolus injection of 35 mL/kg ^2^H_2_O was performed, followed by continuous administration of 8% (volume/volume) ^2^H_2_O in drinking water during specified labeling periods [8, 20]

Oral administration is the only attempted administration route in humans so far, but it may result in delayed or variable tracer absorption, depending on a range of varying factors (*e.g.*, food intake, gastric pH), leading to unstable kinetics [63]. In contrast, IV administration allows for more precise control of the dose and timing of the tracer than oral intake, ensuring uniform distributions of the tracer throughout the body and benefiting a more precise kinetic quantification [63]. Nevertheless, a rapid IV bolus administration might affect the physiology of the body (*e.g.*, changes in blood pressure, heart rate) and alter the intracellular metabolic states in some cases by competing receptors, transporters, and enzymes [31, 72, 77]. Instead, an IV infusion protocol reduces these adverse effects and is particularly useful when studying steady-state metabolism [15, 17]. Still, this infusion protocol has some issues that need to be considered, one of which is the relatively longer time required to reach a real steady-state concentration of the tracer [31, 49, 60]. To achieve a steady state faster, a bolus variable infusion protocol was developed involving a bolus injection of the tracer, followed by a slow but constant infusion [31, 49, 60]. This method may improve the stability and linearity of metabolic quantification during a more than 1-h imaging period [31, 49, 60]. Yet, the bolus variable infusion protocol has challenges that should be considered, such as optimizing variable infusion parameters (*e.g.*, dose, duration, infusion rate). IP administration is another convenient way when performing animal studies [15, 17, 74, 81]. The technique of IP injection is easier than that of IV administration. It allows for the direct delivery of the tracer to the blood circulation through peritoneal absorption. However, this route is probably useless in humans because of the potential risk of bowel injury. Like IV administration, IP administration can also be implemented via a bolus injection or continuous infusion [15, 17, 74, 81].

### Quantitative evaluations of metabolic processes

Before the metabolic quantification, various corrections and normalization procedures should be implemented to obtain accurate molar concentrations of the tracer and metabolites in DMI:

1. Correction for signal variations due to incomplete longitudinal relaxation and RF transmit B_1_+ magnetic field inhomogeneities (the actual RF flip angles) based on measured T1 relaxation times for various metabolites *in vivo* (*e.g.*, water 320 ms, glucose 64 ms, lactate 297 ms, Glx 146 ms, at 11.7 T) and quantitative B1+ maps [15, 29, 37, 50]
2. Normalization of metabolite signals by referring to the naturally abundant semiheavy water signal in the body (about 10.12 mM, acquired before administering tracer as an internal reference), which can automatically correct the inhomogeneity of the receive sensitivity (B1−) of the deuterium RF coil [2, 24, 50, 61, 62, 65]
3. Corrections for deuterium-label loss for each metabolite (*e.g.*, 8.1% label loss for ^2^H-lactate) [15, 50]

In metabolic quantification using DMI, kinetic analysis is most commonly applied. It involves monitoring the blood concentrations of the tracer, the MR signals of the tracers, and the downstream metabolites in each voxel over time, to calculate the flux rate of each target metabolic process in response to different diseases or interventions [37, 44, 47, 48, 66, 83]. So far, IV bolus injection is mostly used in kinetic analysis because it offers a fixed initial concentration of the tracer benefiting the assessment of the flux rate for each metabolite [37, 44, 66]. On the other hand, as discussed above, a bolus injection might introduce neuroendocrine activation and hemodynamic changes, leading to inevitable changes in an individual’s physiological state and metabolic rates [31, 72, 77]. As an alteration, the infusion kinetic analysis reduces the adverse effects caused by tracer administration. It hypothesizes the input rate equals the elimination rate [3, 8, 27, 60, 74]. This method allows for the calculation of stable metabolic rates (*e.g.*, the slope or rate constant) using a relatively simple linear or exponential curve fitting [3, 8, 27, 60, 74].

Besides, there are two other accessible quantification methods: (1) Calculation of the area under the curves of target metabolites over time [24, 28, 29, 61, 69, 73]. A previous study for hyperpolarized-MRI demonstrated the significantly improved reliability of area under the curves-based analysis compared to multiple kinetic analysis, which may shed light as well on the data analysis for DMI [13]. (2) Single time point quantifications [15, 18, 31, 32, 41, 43, 50, 65, 72, 81]. Instead of focusing on dynamic metabolic processes, this method only measured the relative abundance of deuterium-labeled metabolites at a particular time point (commonly after 1 h) [15, 18, 31, 32, 41, 43, 50, 65, 72, 81]. Although it does not provide dynamic information about specific metabolisms, it has been proven as a feasible way to diagnose malignant tumors and evaluate early anti-tumor treatment responses in humans [15]. Compared to kinetic analysis, these two quantification methods have three advantages: simplicity, robustness, and practicability.

### Spatial resolution

The clinical transformation of DMI faces one major challenge: an insufficient spatial resolution with a low signal-to-noise ratio (SNR) [18] (Table 4). Despite the administration of exogenous deuterium-labeled tracers, the concentration of deuterium is still low leading to weak signals. This contrasts with hyperpolarized-^13^C-MRI which provides a higher spatial resolution (0.05 mL *versus* 0.21 mL) with a shorter acquisition time (6 s *versus* 60 s) at 4.7 T than DMI [73]. In a study exploring DMI at 3 T, the spatial resolution reached 3.3 mL with an acquisition time of 10 min [32]. If fixing the acquisition time, spatial resolution and SNR can be improved directly by increasing the B_0_ intensity [18]. A previous study has shown the SNR increased from 169.2 ± 13.3 at 4 T to 423.1 ± 25.7 at 7 T [18]. In animal MR scanners above 9 T, the spatial resolution can reach 0.1 mL with an acquisition time of 5–10 min (Table 4). However, high-field magnet cost and safety issues (*e.g.*, higher radiofrequency energy deposition, dizziness, nausea) limit the clinical translation of DMI [39]. Presently, only 7-T MRI equipment is validated for DMI in clinics, offering a spatial resolution of approximately 1 mL; conversely, this comes with the trade-off of an acquisition time of approximately 30 min (Table 4 and Additional file 1: Table S2) [18]. Although increasing acquisition time is the easiest way to increase spatial resolution by accumulating sufficient signals (Table 4), it introduces the risk of motion artifacts and patient discomfort during the examination. Interpolation and zero-filling are commonly used to enhance spatial resolution, but these enhancements are fake [44, 47].

**Table 4:** A summary of spatial resolution among different studies

Articles	Objects	*B*_0_ field intensity (T)	Spatial resolution (mL)	Repetition time/echo time (ms/ms)	Matrix	Acquisition time (min)
Direct DMI						
[74]	Animals	16.4	0.44	45/NA	9*9*5	1
[41]	Animals	16.4	0.01	NA/NA	17*17*5	1.4^a^
[47]	Animals	15.2	0.15^b^	95/NA	8*8*1	8
[48]	Animals	15.2	0.15^b^	95/NA	8*8*1	8
[56]	Animals	15.2	0.15^b^	95/NA	8*8*1	10
[2]	Animals	14.1	0.11	250/1.35	8*8*1	4
[69]	Animals	14.1	NA	NA/NA	NA	NA
[31]	Animals	11.74	0.016	400/NA	11*11*11	36
[72]	Animals	11.7	0.051	400/0.4	9*9*9	2.5
[17]	Animals	11.7	0.064	400/NA	11*11*11	18
[15]	Animals	11.7	0.064^c^	400/NA	NA	25
[15]	Animals	11.7	0.008^d^	400/NA	NA	35
[72]	Animals	11.7	0.008	400/0.4	15*15*15	37
[46]	Animals	11.1	0.034	100/1.416	32*32*1	13
[37]	Animals	9.4	0.08	140/NA	9*9*3	10
[61]	Animals	9.4	0.25	300/1.25	8*8*1	20
[50]	Animals	9.4	0.016	400/NA	11*11*11	36
[8]^e^	Animals	9.4	NA	NA/NA	NA	NA
[28]	Animals	7	0.08	140/NA	9*9*3	5
[29]	Animals	7	0.081	140/NA	9*9*3	5
[27]	Animals	7	0.4	250/2.3	4*5*1	20.8
[73]	Animals	4.7	0.21	180/NA	8*8*1	6
[62]	Humans	9.4	0.003	155/NA	12*13*14	10
[3]	Humans	7	2	290/1.5	36*36*26	6.5
[65]	Humans	7	2.7	350/NA	14*18*14	28
[18]	Humans	7	1	400/NA	11*11*11	29.5
[43]	Humans	4	8	314/NA	13*9*11	7^g^
[15]	Humans	4	8^d^	333/NA	11*9*9	29
[15]	Humans	4	15.6^c^	333/NA	11*9*10	29
[18]	Humans	4	8	333/NA	11*11*11	29.5
[32]	Humans	3	3.3	120/NA	10*10*10	10
Indirect DMI						
[60]	Animals	9.4	0.03	1500/16^f^	12*12*1	20
[10]	Humans	7	1	2050/40^f^	16*16*1	10
[3]	Humans	7	0.12	320/1.3	36*36*26	3
[52]	Humans	3	0.24	950/NA	32*32*21	4

*DMI* deuterium metabolic imaging, *NA* on-applicable

^a^Average value between 0.9 and 1.8 min

^b^Average value between 0.1 and 0.2 mL

^c^Value was obtained from the liver DMI examinations

^d^Value obtained from the brain DMI examinations

^e2^H-MRI was implemented instead of ^2^H-chemical shift imaging

^f^The Point RESolved Spectroscopy sequence was applied instead of the free induction decay sequence

^g^Interleaved DMI was implemented within the fluid-attenuated inversion recovery acquisition period

Another way to address the spatial resolution or SNR issue in DMI is to improve imaging sequences and coils. The implementation of an Ernst-angle approach, considering an assumed or measured *in vivo* T1, has been shown to enhance signals effectively; for instance, employing a flip angle of 68° for breast cancer in rats with an *in vivo* measured T1 of 250 ms has demonstrated promising results (Table 4) [3, 8, 27, 62]. Another study proposed a multi-echo balanced steady-state free precession approach to enhance SNR (Table 4) [56]. Compared to conventional chemical shift imaging (repetition time/flip-angle = 95ms/90°), the multi-echo balanced steady-state free precession imaging (echo time 2.2 ms, repetition time 12 ms, flip angle 60°) demonstrated a predicted SNR increase of three to five times with matched spatial resolution and scan time, while maintaining good agreement in the time courses of all metabolites [56]. In a previous study, a dual-tuned array coil was introduced, featuring ten transmitting/receiving channels for ^1^H and eight transmitting/two receiving channels for ^2^H, paired with an Ernst-angle three-dimensional chemical shift imaging sequence [62]. This configuration provided a nominal spatial resolution of 0.003 mL with an acquisition time of 10 min and achieved a successful DMI implementation in humans at 7 T (Table 4) [62].

## Discussion

In this work, we provide an extensive overview of the current development of the DMI technique. By analyzing 34 published articles, we introduced and summarized specific technical details and potential applications of DMI *in vivo*. In the following part, we will discuss present limitations, potential research, and development directions of DMI for the future.

So far, direct DMI is still the mainstream technique. While the SNR is constrained by the low gyromagnetic ratio of the deuterium isotope, this limitation is offset by the relatively short T1 of the deuterium-labeled tracer [16, 81]. This characteristic facilitates rapid signal acquisition and allows for a large number of excitations without a significant signal saturation [16, 81]. Compared with direct DMI, indirect DMI has a reported five times higher SNR and can even be performed at commercial 3-T MRI scanners [52, 60]. This eliminates the need for extra spectrometers, specialized deuterium coils, and dedicated MRI sequences. However, from a previous study, the correlation between indirect and direct metabolite quantification was not high (*r* = 0.62) [60]. It indicates the ^1^H-MR signal reduction might be affected by several factors, such as tracer administration methods, stress reaction after tracer administration, participant movement (*e.g.*, participants moved in and out of the scanner to drink the tracer), and so on [31, 72, 77]. After all, the basic hypothesis of indirect DMI is the difference in the absolute metabolite concentrations over time at each pixel. If the absolute metabolite concentrations were affected by tracer administration, the signal reduction would not indicate the real deuterium-labeled metabolite concentrations. Thus, we propose exploring methods to maintain intra-environmental stability of metabolism to address these concerns.

In some cases, the deuteration of certain molecules may affect their metabolism, such as cytochrome P450-mediated oxidative metabolism [4, 64]. Especially, it is essential to address safety considerations in the development of analog tracers [27]. After all, intracellular accumulation of the analog might inhibit glycolysis and bring potential toxicity [38, 80]. However, the deuterated tracers reported in Table 5 rarely interrupt the natural metabolism at the indicated doses and can be safely used *in vivo* [2, 3, 10, 15, 17, 18, 24, 28, 29, 31, 32, 37, 41, 43, 44, 46–48, 50, 52, 56, 60–62, 65, 66, 69, 72–74, 81, 83]. With the help of these different tracers, we can understand the metabolic status of various tissues and organs in living bodies and find the metabolic alterations in various diseases, such as cancer, preeclampsia, and neurological disorders [15, 34, 48, 50]. New development of tracers is still on the way; it is worthwhile to develop tracers on more specific metabolic pathways while prioritizing safety and minimizing label loss.

**Table 5:** A summary of deuterated tracers used *in vivo*

Deuterated tracers	Targeted metabolic pathways	Detected deuterium-labeled metabolites and corresponding chemical shift *in vivo* (ppm)
Deuterated glucose [6,6′-^2^H2]glucose [2, 3, 10, 15, 17, 18, 24, 28, 29, 31, 32, 37, 41, 43, 44, 46–48, 50, 52, 56, 60–62, 65, 66, 69, 72–74, 81, 83], [2,3,4,6,6′-^2^H5]-d-glucose [83], [^2^H7]glucose [46]	Glycolysis; TCA cycle; oxidative phosphorylation	Water (4.8 ppm), glucose (3.8 ppm), Glx (2.4 ppm), Glu4 (2.4 ppm)^a^, lactate (1.4 ppm)
[2,2,2′-^2^H3]acetate [15, 60, 74]	Fatty acid oxidation	Water (4.8 ppm), Glx (2.4 ppm), acetate (1.9 ppm)
[2,3-^2^H2]fumarate [28, 29]	TCA cycle; oxidative phosphorylation	Fumarate (6.5 ppm), water (4.8 ppm), malate (2.4 ppm)
[^2^H9]choline [31, 72]	Choline uptake and following intracellular metabolism	tCho (3.2 ppm)^b^
[U-^2^H]pyruvate [2]	Glycolysis	Pyruvate (2.4 ppm), lactate (1.4 ppm)
Deuterated 3-O-methylglucose [27]	Glucose uptake	Deuterated 3-O-methylglucose (3.5 ppm)
^2^H_2_O [8]	Deoxyribonucleic acid synthesis	Deuterium signal^c^

*Glu4* [4,4′-^2^H2]glutamate and [4′-^2^H]glutamate, *Glx* Glutamine and glutamate, *TCA* Tricarboxylic acid, *tCho* Total choline pool

^a^Instead of a single Glx peak in direct DMI, individual distinction of Glu4 from glutamine can be achieved by indirect DMI as mentioned above [60]

^b^Deuterated choline cannot be identified from its downstream metabolites such as phosphocholine and glycerophosphocholine under magnetic resonance spectroscopy because of the spectral overlap; thus, the deuterium-labeled pool of choline plus metabolites was quantified

^c^No chemical shift imaging or spectroscopy was performed in this study; instead, non-selective deuterium-nuclear MRI was applied with the dual transmit/receive coil tuned to 61.45 MHz [8]

Overall, DMI holds potential for various medical applications in the future, including cancer diagnosis, early response evaluation after anti-tumor treatment, nutritional studies, and metabolic disease research. As another deduction that merits further exploration, DMI might also help identify the “pseudoprogression” after immune checkpoint inhibitor therapies, characterized by an initial increase followed by a subsequent decrease in the size of existing tumors after treatment [22, 40]. In addition, due to the non-radioactive feature, DMI presents a potential utilization in human fetuses and pregnant women, although definitive evidence is yet to be established; however, the feasibility of DMI has been demonstrated in pregnant mice [48].

There are different administration routes in DMI, but the comparisons between varied routes *in vivo* are still limited. After all, the different routes of administration may affect the plasma glucose level and the deuterium enrichment of downstream metabolites, which can influence the sensitivity and accuracy of DMI. One previous study explored the discrepancies between IV and IP infusions of [6,6′-^2^H2]glucose and found that IV infusions caused the glucose signal to rapidly reach a plateau in the liver, while IP infusions showed a continuous rise in the glucose signal, far surpassing the water signal [17]. Another study comparing IV bolus injection and infusion in mice found that while infusion led to a smoother glucose response without an initial spike, minimal differences were observed in downstream lactate metabolite quantification compared to bolus injection [48]. So far, while the emphasis on oral administration for humans has stemmed from its perceived safety, simplicity, and well-tolerance, alternative administration routes have not been extensively explored rather than being deemed inherently infeasible. Therefore, for clinical translation, it is essential to comprehensively compare different tracer administration routes in further investigations.

Although kinetic analysis is the most commonly applied quantification in DMI, it still has several challenges. First, it requires multiple time point measurements, which can be time-consuming and resource-intensive. Second, it requires complex mathematical modeling to calculate kinetic parameters, requiring a sufficiently short acquisition time of DMI. Third, even the infusion kinetic analysis may lead to inaccurate estimation of metabolic rates because the tracer is always compartmentalized within specific tissues or metabolic pathways resulting in a very complex kinetic system [3, 8, 27, 60, 74]. To tackle these issues, quantification methods, including calculation of the area under the curves of target metabolites over time and single time-point quantifications, can be considered [15, 18, 24, 28, 29, 31, 32, 41, 43, 50, 61, 65, 69, 72, 73, 81]. However, these two methods are limited because they may not capture the dynamic information as effectively as kinetic modeling. Overall, future research is likely to focus on addressing the challenges associated with kinetic analysis while exploring alternative quantification methods to enable more accurate and efficient assessment of metabolic processes in clinical scenarios.

To date, the clinical transformation of DMI heavily hinges on addressing the limitation of spatial resolution and SNR. Enhancing deuterium enrichment of tracers (*e.g.*, [2,3,4,6,6′-^2^H5]glucose and [^2^H7]glucose) and applying the indirect DMI strategy are both potentially applicable to enhance SNR for DMI, as introduced in previous sections (Table 4 and Additional file 1: Table S2), but these advancements have yet to fully overcome existing challenges [3, 46, 52, 60, 83]. Recently, deep learning techniques have offered a promising avenue for DMI denoising, resulting in improved SNR. In a pioneering study, researchers proposed a novel machine learning-based approach that synergistically integrates physics-based subspace modeling and data-driven deep learning to achieve effective denoising, enabling high-resolution DMI [41]. In addition, a less explored technique in DMI is compressed sensing, which may hold the potential to reduce the acquisition time while preserving data quality by leveraging the inherent sparsity of metabolic signals in a relatively wide spectrum, meriting further investigations [19, 21].

In conclusion, DMI holds promise for improving clinical diagnostics and treatment protocols by offering new insights into metabolic disorders and diseases. Despite significant advancements, limitations in spatial resolution still hinder the clinical translation of DMI techniques. Additionally, to unlock the full clinical potential of DMI, it is essential to optimize tracer synthesis, administration protocols, and quantitative analysis methodologies.

### Supplementary Information

**Additional file 1:** **Table S1.** Search strategy. **Table S2.** Comparisons of spatial-temporal resolution among different studies.

## Data Availability

The authors confirm that the data supporting the findings of this study are available within the article and its supplementary materials.

## Abbreviations

DMI: Deuterium metabolic imaging
Glx: Glutamine and glutamate
IP: Intraperitoneal
IV: Intravenous
MR: Magnetic resonance
MRI: Magnetic resonance imaging
MRS: Magnetic resonance spectroscopy
MRSI: Magnetic resonance spectroscopic imaging
SNR: Signal-to-noise ratio
TCA: Tricarboxylic acid
