# Supplementary material for: Advances and prospects in deuterium metabolic imaging (DMI): a systematic review of in vivo studies
Source: Eur Radiol Exp. 2024 Jun 3;8:65. doi: 10.1186/s41747-024-00464-y (PMC11144684; doi:10.1186/s41747-024-00464-y)
Supplement: Supplementary file 1 — Additional file 1: Table S1. Search strategy. Table S2. Comparisons of spatial-temporal resolution among different studies. [file 41747_2024_464_MOESM1_ESM.pdf]

Advances and prospects in deuterium metabolic imaging (DMI): a systematic review of in vivo studies

ELECTRONIC SUPPLEMENTARY MATERIAL

Supplementary Table S1. Search strategy

Search date	Search strategy
24th May 2023	("deuterium"[MeSH Terms] OR "deuterium"[All Fields] OR "deuteriums"[All Fields]) AND ("magnetic resonance imaging"[MeSH Terms] OR ("magnetic"[All Fields] AND "resonance"[All Fields] AND "imaging"[All Fields]) OR "magnetic resonance imaging"[All Fields] OR ("magnetic resonance spectroscopy"[MeSH Terms] OR ("magnetic"[All Fields] AND "resonance"[All Fields] AND "spectroscopy"[All Fields]) OR "magnetic resonance spectroscopy"[All Fields])) AND ("2003/05/24"[PDat] : "2023/05/24"[PDat])

Supplementary Table S2. Comparisons of spatial-temporal resolution among different studies.

DMI techniques	Objects	B0 field intensity (Tesla)*	Spatial resolution (ml)*	Temporal resolution (minutes)*
Direct DMI	Animals	11.4 ± 3.3<br>(4.7-16.4)	0.119 ± 0.124<br>(0.008-0.440)	15.1 ± 12.6<br>(1.0-37.0)
	Humans	5.5 ± 2.2<br>(3.0-9.4)	5.400 ± 4.951<br>(0.003-15.600)	19.8 ± 10.9<br>(6.5-29.5)
	Total	9.7 ± 4.0<br>(3.0-16.4)	1.758 ± 3.633<br>(0.003-15.600)	16.6 ± 12.1<br>(1.0-37.0)
Indirect DMI	Animals	9.4	0.030	20.0
	Humans	5.7 ± 2.3<br>(3.0-7.0)	0.453 ± 0.477<br>(0.120-1.000)	5.7 ± 3.8<br>(3.0-10.0)
	Total	6.6 ± 2.7<br>(3.0-9.4)	0.348 ± 0.443<br>(0.030-1.000)	9.3 ± 7.8<br>(3.0-20.0)

Abbreviation: DMI, deuterium metabolic imaging.

Note: *After summarizing the data in Table 5, all results were presented as mean ± standard deviation (minimum-maximum).
